# Enhancement of catalytic activity by UV-light irradiation in CeO_2_ nanocrystals

**DOI:** 10.1038/s41598-019-44543-2

**Published:** 2019-05-29

**Authors:** Tai-Sing Wu, Leng-You Syu, Chao-Nan Lin, Bi-Hsuan Lin, Yi-Hsiu Liao, Shih-Chang Weng, Yuh-Jeen Huang, Horng-Tay Jeng, Shih-Yuan Lu, Shih-Lin Chang, Yun-Liang Soo

**Affiliations:** 1 Department of Physics, National Tsing Hua University, Hsinchu, Taiwan; 2 Department of Biomedical Engineering and Environmental Sciences, National Tsing Hua University, Hsinchu, Taiwan; 3 National Synchrotron Radiation Research Center, Hsinchu, Taiwan; 4 Department of Chemical Engineering, National Tsing Hua University, Hsinchu, Taiwan; 5 Institute of Physics, Academia Sinica, Taipei, Taiwan

**Keywords:** Chemical physics, Electrocatalysis, Catalytic mechanisms

## Abstract

Ultraviolet (UV) light irradiation on CeO_2_ nanocrystals catalysts has been observed to largely increase the material’s catalytic activity and reactive surface area. As revealed by x-ray absorption near edge structure (XANES) analysis, the concentration of subvalent Ce^3+^ ions in the irradiated ceria samples progressively increases with the UV-light exposure time. The increase of Ce^3+^ concentration as a result of UV irradiation was also confirmed by the UV-vis diffuse reflectance and photoluminescence spectra that indicate substantially increased concentration of oxygen vacancy defects in irradiated samples. First-principle formation-energy calculation for oxygen vacancy defects revealed a valence-hole-dominated mechanism for the irradiation-induced reduction of CeO_2_ consistent with the experimental results. Based on a Mars-van Krevelen mechanism for ceria catalyzed oxidation processes, as the Ce^3+^ concentration is increased by UV-light irradiation, an increased number of reactive oxygen atoms will be captured from gas-phase O_2_ by the surface Ce^3+^ ions, and therefore leads to the observed catalytic activity enhancement. The unique annealing-free defect engineering method using UV-light irradiation provides an ultraconvenient approach for activity improvement in nanocrystal ceria for a wide variety of catalytic applications.

## Introduction

Ceria-containing catalysts have attracted considerable research interests due to their remarkable catalytic activity in a variety of important chemical reactions^1–9^. It is well-known that Ce^3+^ and Ce^4+^ ions coexist in ceria and the high catalytic activity has been largely attributed to the Ce^4+^/Ce^3+^ redox cycle that facilitates effective uptake and release of oxygen atoms on the ceria surface. Based on a widely accepted Mars-van Krevelen mechanism for ceria catalyzed oxidation processes, the reactant is mainly oxidized by the oxygen atoms captured from gas-phase O_2_ by the Ce^3+^ ions on the catalyst’s surface. Therefore, the concentration of Ce^3+^ ions on ceria surface plays a pivotal role in determining the catalyst’s activity. To further enhance the activity of CeO_2_, the size, shape, morphology and defects of the materials have been manipulated to expose the most Ce^3+^-rich facets^2–6^ or increase the concentration of oxygen vacancy defects, that give rise to Ce^3+^ ^3,7,8^. As a defect engineering method for activity enhancement, thermal annealing in low-pressure or reductive atmosphere is often used to create oxygen vacancy defects in ceria samples. However, agglomeration of nanoparticles that usually occurs during a thermal annealing process can severely reduce the surface to volume ratio and therefore pose a serious problem for the activity enhancement. On the other hand, irradiation of ceria by ionizing radiation such as UV-light may break Ce-O bonds to generate oxygen vacancy defects and therefore increase the catalyst’s activity due to increased Ce^3+^ concentration. In the present work, we demonstrate an annealing-free method for creating oxygen vacancy defects in CeO_2_ nanocrystals by using UV-light irradiation.

**Figure 1:**
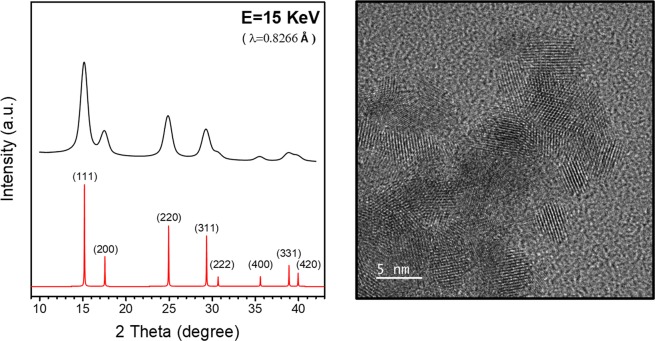
XRD patterns and a TEM micrograph of the as-made CeO_2_ sample.

**Figure 2:**
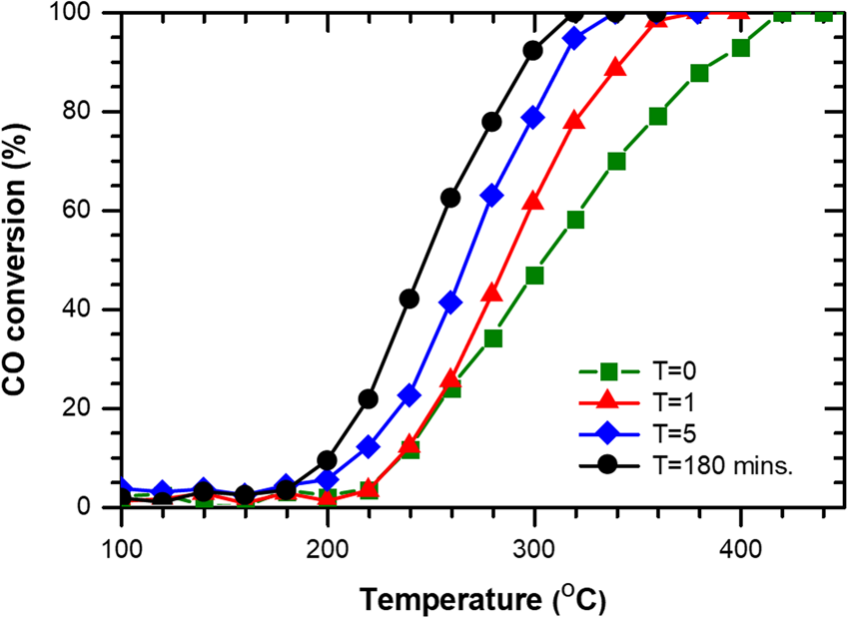
Comparison of the catalytic activity of the as-made CeO_2_ sample with those of samples irradiated by UV light for various irradiation time.

Nanocrystal samples of CeO_2_ were prepared using a polyol method^10^. As shown in Fig. 1, the synchrotron-based x-ray powder diffraction (XRD) data for the as-made sample match well with that of cubic CeO_2_ at the (111), (200), (220), (311), (222), (400), (331) and (420) Bragg peaks. The crystallite size determined by using Scherrer equation is 4.4 nm, which is consistent with that estimated from the TEM micrograph. The TEM micrograph also indicate high crystallinity and rather uniform particle size of the nanocrystals. The as-made samples were then irradiated by UV-light in N_2_ for various exposure time, using a homemade UV-light device. The photon energy of the UV-light source is 4.88 eV (254 nm, UVC), larger than the 3.0 eV bandgap value of CeO_2_. The power of UV light on the sample is around 7.5 mw/cm^2^. Brunauer-Emmett-Teller (BET) surface area and CO oxidation reaction measurements were used to compare the reactive surface area and the catalytic activity of the as-made sample with those of the UV-irradiated samples, respectively. The BET surface areas of the samples were measured by using a Micromeritics ASAP 2020 surface area analyzer with nitrogen. After 180 minutes of UV-light irradiation, the BET surface area increased from 54.9 to 72.4 m^2^/g. With the increase of Ce^3+^ concentration due to UV irradiation, the surface of nanoparticles becomes more reactive and therefore is able to absorb more gas molecules. The reaction ${\rm{CO}}+1/2{{\rm{O}}}_{2}\to {{\rm{CO}}}_{2}$ was monitored by quantifying the concentration of the effluent gas with a gas chromatograph (GC) device equipped with a thermal conductivity detector. The catalyst weight was 30 mg and the total flow rate of the reaction gas was 100 SCCM, with a composition of 5% CO– 25% O_2_ (balanced with He gas). The conversion of CO was calculated from the CO concentrations in the inlet and outlet gases. It is clear that UV-light irradiation progressively enhances the catalytic activity of the sample as the irradiation time increases from 0 to 180 minutes (Fig. 2). The light-off temperature T_50_ decreased from 305 to 248 °C and the turnover number (TON) at 260 °C increased from 2.78 to 7.17 ${\mu }\mathrm{mol}\cdot {g}^{-1}\cdot {s}^{-1}$ after 180 minutes of UV-light irradiation on the as-made sample.

**Figure 3:**
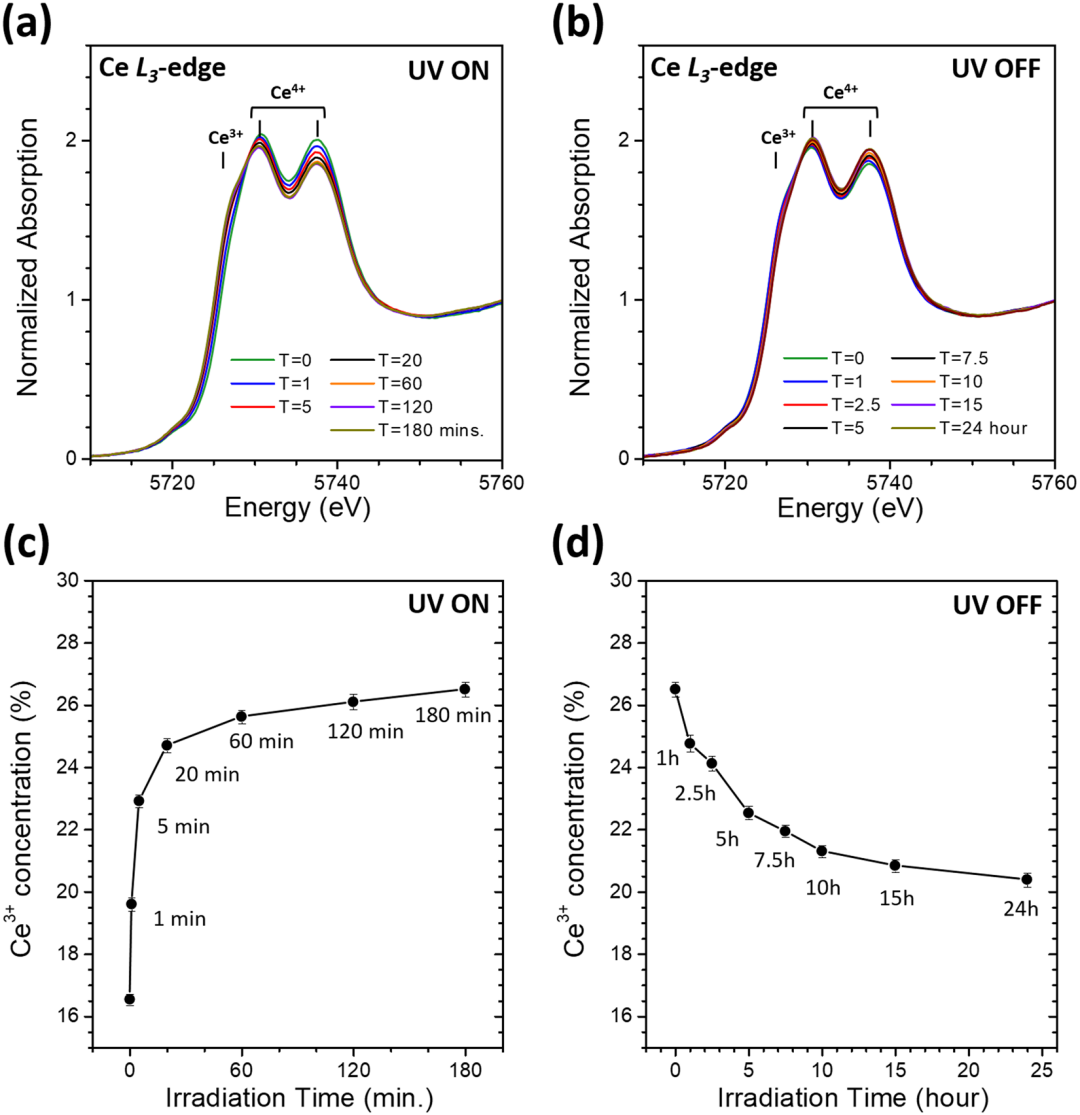
(**a**) Ce L3-edge XANES data of the as-made CeO_2_ sample irradiated by UV-light for different time durations. (**b**) Ce L3-edge XANES data of the 180-min-irradiated CeO_2_ sample after being stored in ambient condition with UV-light off for different time durations. (**c**) A plot of Ce^3+^ concentration vs. UV-light irradiation time of the as-made CeO_2_ sample. (**d**) A plot of Ce^3+^ concentration vs. UV-light-off time of the 180-min-irradiated CeO_2_ sample.

To obtain experimental evidence for the proposed mechanism that UV-light irradiation induces reduction of Ce^4+^ ions and hence gives rise to catalytic activity enhancement in these ceria samples, Ce L_3_-edge x-ray absorption near edge structure (XANES) was measured to monitor the evolution of peaks ascribed to Ce^3+^ and Ce^4+^, as well as the Ce^3+^ concentration calculated therefrom. The XANES measurements were performed at beamline 07A of the Taiwan Light Source (TLS) at National Synchrotron Radiation Research Center (NSRRC) in Taiwan. A Si(111) double-crystal monochromator was used to scan the photon energy of the incident x-ray beam and the estimated energy resolution (ΔE/E) was 2.0 × 10^−4^. The conventional transmission mode of detection was adopted for the measurements of all samples. The raw experimental XANES data for all samples are shown in Fig. 3(a).

To extract Ce^3+^ concentration from XANES data, the spectra were curve-fitted with an arctangent function to simulate the edge jump and Gaussian functions for peak features. The center of the arctangent function was set at the inflection point of the main edge. The peaks at 5737.7 eV and 5730.8 eV were associated with the Ce^4+^ ions in ceria while the peak at 5726.3 eV was due to the Ce^3+^ valence state. The concentration of Ce^3+^ in the samples were calculated using the following equations:

$$[{{\rm{Ce}}}^{3+}]={\rm{A}}({{\rm{Ce}}}^{{\rm{3}}+})/({\rm{A}}({{\rm{Ce}}}^{{\rm{3}}+})+{{\rm{A}}(\mathrm{Ce}}^{{\rm{4}}+}))$$

where A(Ce^3+^) and A(Ce^4+^) were the total integrated peak areas corresponding to the Ce^3+^- and Ce^4+^-associated peaks, respectively. More details on the curve-fitting method used for extracting Ce^3+^ concentration from XANES spectra has been reported elsewhere^11^. As shown in Fig. 3(a), the peak ascribed to Ce^3+^ progressively increases with the exposure time. Figure 3(c) also demonstrates that the Ce^3+^ concentration obtained from curve-fitting the XANES spectra increases rapidly from 16.5 at. % and saturates at 26.5 at. % as the exposure time increases.

**Figure 4:**
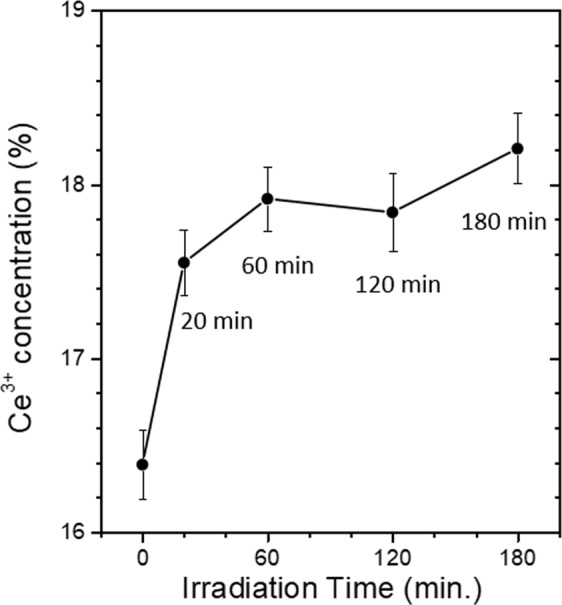
A plot of Ce^3+^ concentration vs. UV-light irradiation time of the as-made CeO_2_ sample irradiated in a pure O_2_ atmosphere.

To investigate the stability of this defect engineering technique, the sample with saturated Ce^3+^ concentration was then stored in ambient condition with UV-light off for 24 hours. As shown in Figs 3(b,d), the Ce^3+^ concentration slowly decreases from 26.5 at. % to 20.4 at. % during this period of time, indicating that the Ce^3+^ rich sample can remain relatively stable for hours. Furthermore, to better understand the oxygen escaping mechanism, the UV-light irradiation treatment was also carried out in an oxygen-rich atmosphere. As demonstrated in Fig. 4, after 180 minutes of UV-light irradiation on the as-made CeO_2_ sample in a pure O_2_ atmosphere, the Ce^3+^ concentration of the sample increases from 16.5 at. % and saturates at a much lower value of 18.2 at. % compared to that of irradiation in N_2_. The reduction effect of UV-irradiation on the sample may be partially neutralized due to re-oxidation of the sample by oxygen in the pure O_2_ atmosphere.

**Figure 5:**
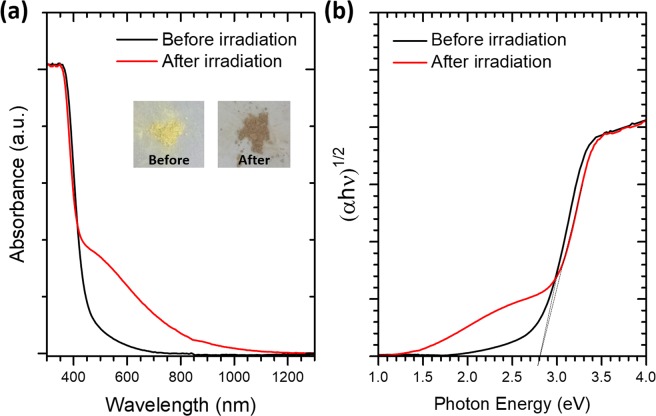
(**a**) UV-vis diffuse reflectance spectrum of ceria nanoparticles and (**b**) the Tauc plot (αhν)^1/2^ vs (hν) for CeO_2_ sample before and after 3 hours of UV-light irradiation.

**Figure 6:**
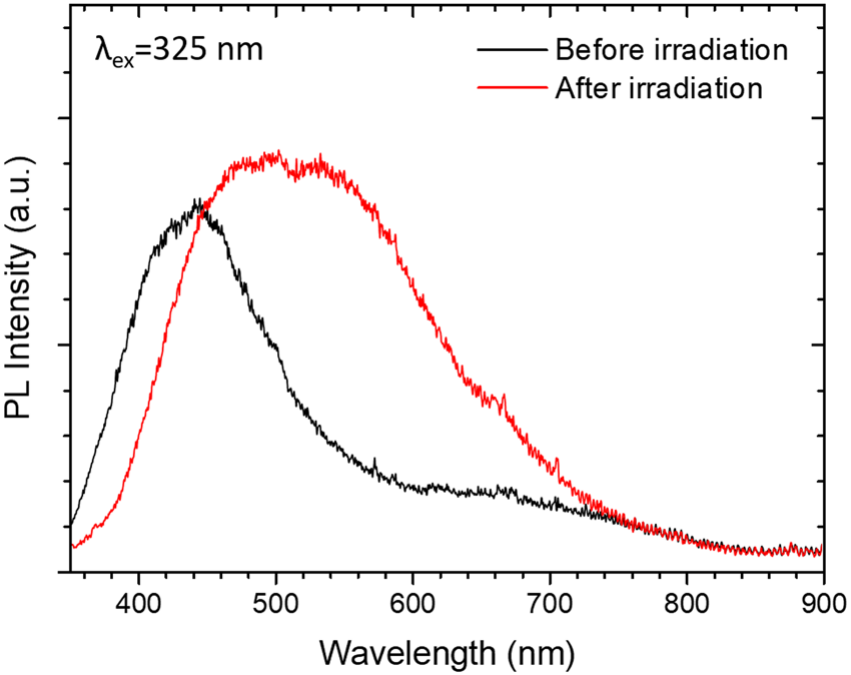
Room temperature photoluminescence (PL) spectra of as-made and UV-light irradiated CeO_2_ nanocrystal sample at an excitation wavelength of 325 nm.

Ultraviolet-visible (UV-vis) diffuse reflectance spectroscopy and photoluminescence (PL) measurements have also been performed to provide additional evidence for the increased number of oxygen vacancy defects due to UV-light irradiation in the ceria sample. As shown in Fig. 5(a), the UV-vis spectrum after 3 hours of UV light irradiation indicates heightened absorption in the visible light region compared to that before irradiation. Consequently, the color of the sample appeared to be severely darkened after 3 hours of UV-light irradiation. It is conceivable that the increased concentration of oxygen vacancy defects as a result of UV irradiation can effectively enhance the absorption of visible light due to transitions related to defect levels and therefore give rise to the darkening of sample color. The band gap values of the samples were determined by fitting the UV-vis data with Kubelka-Munk function^12^ and Tauc’s plots^13^, as shown in Fig. 5(b). The band gap width for the as-made sample 2.8 eV is smaller than the reported indirect band gap of bulk ceria in the range between 3.0 and 3.2 eV. Such band-gap narrowing effect is most likely due to the defect states near the bottom of conduction band which are formed by oxygen vacancies and Ce^3+^ of the nanocrystals^14^. After UV-light irradiation, the oxygen vacancy defects proliferate, giving rise to an increased number of defect-related intermediate states that further combine to form a new band at lower energy in the original band gap. The photoluminescence (PL) spectra measured at room-temperature by using an excitation source of wavelength 325 nm are shown in Fig. 6. For the as-made sample, the PL spectrum exhibits a strong peak at around 440 nm (2.8 eV), attributed to the transition from Ce 4 f to the valance band. After UV irradiation, a large number of oxygen vacancy defects were created. The direct transition of electrons from Ce 4 f to the valance band is retarded due to the presence of defect-related intermediate states. A broad emission peak associated with oxygen vacancy defects appears at 500 nm (2.5 eV)^14^. The UV-vis and PL data both indicate increased oxygen vacancy defects in the irradiated samples and thus provide additional evidence that confirms the XANES results of Ce^3+^ concentration increase due to UV-light irradiation.

**Figure 7:**
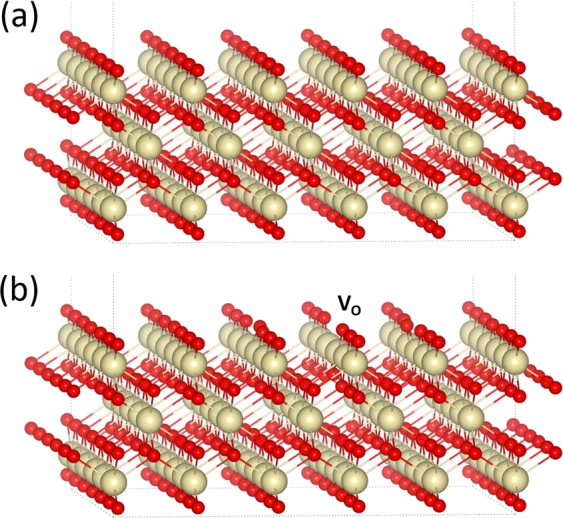
(**a**) Supercell of CeO_2_ (100) slab used in the calculation. (**b**) Schematic view of CeO_2_ (100) slab with one oxygen vacancy defect. The large white balls and small red balls represent the Ce and O atoms, respectively.

The experimentally observed effect of ceria reduction by UV-light irradiation was also theoretically investigated by first-principle spin-polarized density functional theory (DFT) calculation using the VASP code^15^. The projector augmented-wave (PAW) method and the generalized gradient approximation (GGA)/Perdew-Burke-Ernzerhof (PBE) functional were adopted in the calculation^16^. For the corrections of on-site Coulomb interactions, we have used a GGA + U method with U = 5 eV applied to the Ce 4*f*-states^17,18^. The Ce(5s^2^5p^6^6s^2^4f^1^5d^1^), and O(2s^2^2p^4^) valence states were expanded in a plane-wave basis with a kinetic-energy cutoff of 400 eV. A periodic slab model, based on a 19.13 Å × 19.13 Å × 19.13 Å supercell that contains an 8 Å-thick crystal slab and an 11 Å-thick vacuum layer, was used as the initial structure in the calculation, as shown in Fig. 7(a). The 8 Å-thick crystal slab is composed of 7 atomic layers of a (5 × 5) expansion of the CeO_2_ (100) surface and is terminated by an oxygen layer on the top and bottom, where half of the oxygen atoms have been removed from each terminating oxygen layer. A total of 225 atoms (75 CeO_2_ units) are included in the supercell. During structure optimization, the atomic coordinates of all atoms, except for those on the bottom atomic layer of a slab, were allowed to vary until the maximum force on each atom was smaller than 0.02 eV/Å. The Brillouin zone was sampled using a 4 × 4 × 1 Monkhorst–Pack (MP) grid. The effect of UV-light irradiation was represented by the creation of valence holes in the slab model. As shown in Fig. 7(b), an O atom was removed from the surface of slab to mimic the formation of an O vacancy. The formation energies of the surface O vacancy ${E}_{VO}^{F}$ were obtained as the difference in total energies between two supercells using the following equation:

$${E}_{VO}^{F}(q)={E}_{T}(C{e}_{75}{O}_{149},q)-{E}_{T}(C{e}_{75}{O}_{150},q)+\frac{1}{2}{E}_{T}({O}_{2})$$

where ${E}_{T}(C{e}_{75}{O}_{149},q)$ and ${E}_{T}(C{e}_{75}{O}_{150},q)$ are the total energies of the optimized supercells with and without an surface O vacancy, respectively, *q* is the number of electron removed from the supercell, and ${E}_{T}({O}_{2})$ is the total energy for the triplet ground state of an optimized oxygen molecule in the gas phase.

The surface O vacancy formation energy of CeO_2_ (100) was calculated to be 1.16 eV, close to the value reported previously^19^. When one valence electron is removed as a result of UV-light irradiation, the O vacancy formation energy decreases to 0.44 eV. As an O atom is removed from CeO_2_, the two electrons left on the O vacancy site will be transferred to the two nearest Ce atoms to form Ce 4*f*^1^ defect states^17,18,20^. In the presence of a valence hole created by UV-light irradiation, only one electron is to be transferred and the Coulomb energy of the system is thus largely lowered. When the UV-light irradiation creates two valence holes, the O vacancy formation energy is further lowered to a negative value of −0.64 eV, representing the onset of O vacancy formation in CeO_2_. As oxygen vacancies are generated by UV-light irradiation, the Ce^3+^ concentration in the ceria catalyst is increased. We have therefore theoretically demonstrated the reduction mechanism of CeO_2_ induced by valence-hole-generating UV-light irradiation.

We note that a similar procedure for calculating O-vacancy formation energies was reported in one of our previous papers in which the mechanism for defect engineering using x-rays in nanocrystal ceria was investigated in details^21^. However, as opposed to x-rays that penetrate deep into the irradiated materials, UV irradiation is mostly effective near the surface of the materials. Therefore, the supercell model used in our previous paper was replaced by a slab model in the present work. As a result, the calculated formation energies are substantially lowered compared to those in our previous paper.

It’s also worth noting that UV-light irradiation during catalyzed reactions has previously been applied to improve the reaction rates, such as those in the photocatalytic effect of metal-semiconductor system with hot electrons transferring from metal surface to semiconductor^22^ and the synergetic effect that occurs at the interface of photocatalyst and thermal catalyst^23^. By contrast, our unprecedented defect engineering approach using UV-light irradiation treats the ceria catalysts before using ceria to catalyze the chemical reactions.

In conclusion, we have experimentally demonstrated that UV-light irradiation can generate a large number of oxygen vacancy defects in CeO_2_ and therefore achieve effective reduction of ceria. Theoretical DFT calculation of the formation energies for oxygen vacancy defects in all samples reveals a valence-hole-dominated mechanism for irradiation-induced oxygen vacancy formation consistent with the experimentally observed results. As the oxygen vacancy defects are generated, and thus Ce^3+^ concentration is increased, by UV-light irradiation, more efficient oxidation of reactants, and therefore enhanced catalytic activity of ceria, are achieved, based on the Mars-van Krevelen mechanism for the ceria catalyzed oxidation processes. The improved catalytic activity observed by CO conversion measurements for the irradiated samples indicates that UV-light irradiation is an effective and annealing-free method to enhance ceria activity for important catalytic applications.

## Methods

Ceria nanocrystal samples were prepared using a polyol method. The crystal structures and particle sizes of the samples were determined by synchrotron-based XRD using the Scherrer equation. The TEM micrographs were also used to obtain further information on the crystallinity and particle size of the nanocrystals. The as-made samples were irradiated by the 254 nm UV-light in N_2_ and O_2_ for various exposure time. Brunauer-Emmett-Teller (BET) surface area and CO oxidation reaction measurements were used to obtain the reactive surface area and the catalytic activity of the samples. The Ce L_3_-edge XANES performed at beamline 07A of the Taiwan Light Source at NSRRC in Taiwan, was employed to monitor the evolution of Ce^3+^ concentration due to UV irradiation in the samples. The UV-vis diffuse reflectance spectroscopy and PL measurements have also been performed to provide additional evidence for the variation of oxygen vacancy defects due to UV-light irradiation in the ceria sample. Finally, the experimentally observed effects of UV-light irradiation on ceria was theoretically investigated by first-principle DFT calculation using the VASP code.

## Data Availability

All data generated or analysed during this study are included in this published article.
